# A Power-Efficient Radiation Sensor Interface with a Peak-Triggered Sampling Scheme for Mobile Dosimeters

**DOI:** 10.3390/s20113255

**Published:** 2020-06-07

**Authors:** Hyuntak Jeon, Injun Choi, Soon-Jae Kweon, Minkyu Je

**Affiliations:** School of Electrical Engineering, Korea Advanced Institute of Science and Technology (KAIST), Daejeon 34141, Korea; ht.jeon@kaist.ac.kr (H.J.); injunchoi@kaist.ac.kr (I.C.); mkje@kaist.ac.kr (M.J.)

**Keywords:** radiation sensor interface, silicon photomultiplier (SiPM), mobile dosimeter, analog-to-digital converter (ADC)

## Abstract

Radiation sensor interfaces for battery-powered mobile dosimeters must consume low power to monitor the amount of radiation exposure over a long period. This paper proposes a power-efficient radiation sensor interface using a peak-triggered sampling scheme. Since the peak of the analog-to-digital converter’s (ADC’s) input represents radiation energy, our ADC only operates around the peak value thanks to the proposed sampling scheme. Although our ADC operates with a high sampling frequency, this proposed sampling scheme reduces the power consumption of the sensor interface because of the reduced operation time of the ADC. Our sensor interface does not have signal distortion caused by a conventional shaper because the interface quantizes the peak value using the high sampling frequency instead of the shaper. When the radiation input occurs once every 10 μs, the power consumption of the ADC with the proposed sampling scheme is only about 21.5% of the ADC’s power consumption when the ADC continuously operates. In this worst case, the fabricated radiation sensor interface in a 0.18-μm complementary metal-oxide-semiconductor (CMOS) process consumes only 1.11 mW.

## 1. Introduction

Nuclear power plants have been the basis of modern industrial development because these plants produce large amounts of highly efficient electricity. However, since the recent Fukushima nuclear accidents, concerns about the safety of nuclear power plants have been raised more than before. The radiation exposure over a certain level may cause biologically harmful effects, such as carcinogenesis [1,2,3]. As a result, not only the nuclear power plant workers managed by competent agents, but also the ordinary people in daily life are at increased risk of radiation exposure [4].

**Figure 1:**
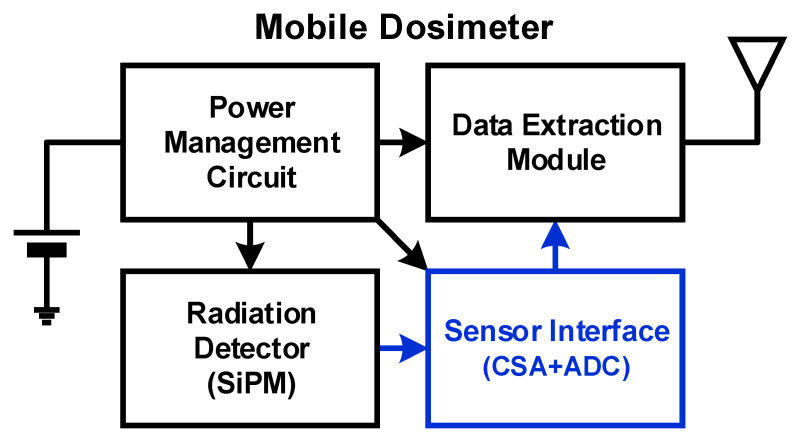
Block diagram of a mobile dosimeter.

If people in daily life can easily use mobile dosimeters, they will be able to promptly address harmful situations. Figure 1 shows a block diagram of a mobile dosimeter, which consists of a radiation detector, a sensor interface, a data extraction module, and a power management circuit. Because the required bias voltage of silicon photo-multipliers (SiPMs) is lower than that of other radiation detectors [5], SiPMs are currently being used as radiation detectors. The sensor interface converts the output of the radiation detector to digital outputs, and the data extraction module processes these digital outputs appropriately for transmitting data to data servers or other mobile devices. The power management circuit generates supply voltages of the mobile dosimeter from a small battery for users to make these dosimeters portable in daily life. In such a battery-powered system, it is important to implement each subsystem in a power-efficient manner for a long time of operation. We propose a power-efficient radiation sensor interface because prior ones consume high power in handling high-speed radiation signals.

## 2. Prior Radiation Sensor Interface

**Figure 2:**
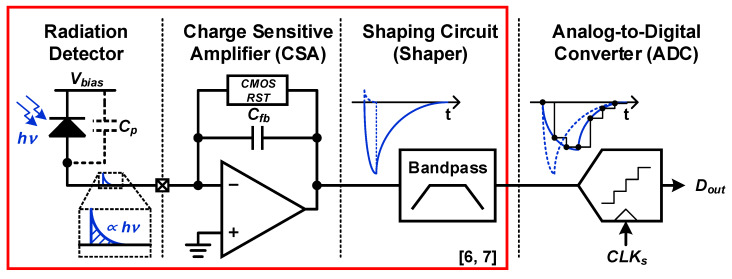
Architecture and operation of prior radiation sensor interfaces.

Figure 2 shows the architecture and operation of prior radiation sensor interfaces [6,7,8,9,10,11]. It consists of a charge-sensitive amplifier (CSA), a shaper, and an analog-to-digital converter (ADC). The radiation detector converts radiation energy (*hν*) into charge signals. When radiation particles are injected into the radiation detector, charges are generated in the form of spikes, which have a short period. The accumulated charge amount is proportional to the radiation energy. 

The CSA integrates the charge spike into the feedback capacitor to generate the voltage signal, which has enough amplitude for the ADC to handle [6,7,11]. Since the parasitic capacitance (*C_p_*) of the radiation detector is much larger than the feedback capacitance (*C_fb_*), the voltage loop gain of the CSA is close to zero, and the integration is performed through the open-loop bandwidth of the operational amplifier (OP-AMP). As a result, the conversion from charge to voltage through this type of CSA is much more power-efficient than the conversion through closed-loop amplifiers. In this CSA, the feedback capacitor must be periodically reset through a complementary metal-oxide-semiconductor (CMOS) switch and other digital logic elements to recover the original DC bias point of OP-AMP before integrating the newly generated charge spike. 

As shown in Figure 2, since the peak of the CSA’s output represents radiation energy, the ADC must quantize the peak value of the CSA’s output. When the CSA’s output is directly quantized by the ADC, the ADC needs a very high sampling frequency (*f_s_*) due to the short period of the charge spike. In order to reduce *f*_s_, the shaper is introduced to stretch the period of the CSA’s output. When the ADC quantizes the shaper’s output rather than the CSA’s output, the ADC can quantize the peak value of the CSA’s output with a lower *f_s_*. This results in reducing the power consumption of the ADC. However, the shaper, which has a bandpass characteristic, causes signal distortion because the charge spike itself has a wideband characteristic inherently.

## 3. Proposed Radiation Sensor Interface

**Figure 3:**
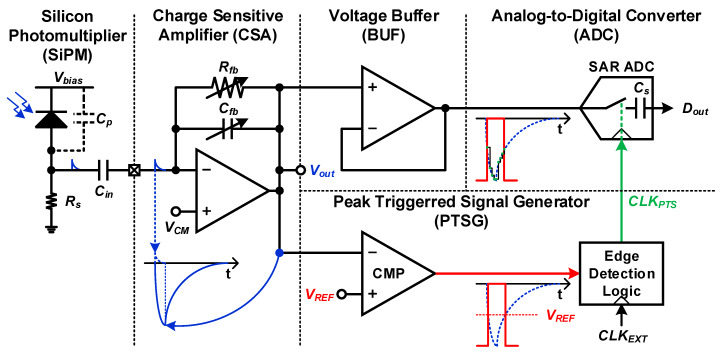
Architecture and operation of the proposed power efficient radiation sensor interface.

We propose a peak-triggered sampling scheme that minimizes the power consumption of the ADC and quantizes the peak without the shaper. Figure 3 shows a block diagram of the proposed radiation sensor interface for the mobile dosimeter. The sensor interface consists of a CSA, a proposed peak-triggered signal generator (PTSG), and a successive approximation register (SAR) ADC with a voltage buffer (BUF). The CSA converts charge spikes to voltage signals, and the SAR ADC quantizes the CSA’s output using a sampling clock signal (*CLK_PTS_*), which is generated by the PTSG. Since this *CLK_PTS_* has logic transitions only around the peak of the CSA’s output (*V_out_*), the PTSG reduces the operation time of the ADC. The BUF is adopted to precisely sample *V_out_* on a large sampling capacitor of the SAR ADC. A more detailed description of operation is as follows.

Our sensor interface supports a radiation detector based on SiPM with a series resistor and a DC blocking capacitor. The DC blocking capacitor with a capacitance of 1 μF is used to decouple the DC bias points of the SiPM output and the CSA input. We also adopt the open-loop CSA used in [6,7] to convert the charge spike to voltage signals with low power consumption. Instead of the reset switch in Figure 2, our CSA maintains DC operating point through a feedback resistor (*R_fb_*) not to miss charge spikes without any complicated digital logic elements [12].

The proposed PTSG, which consists of a comparator (CMP) and an edge detection logic, generates *CLK_PTS_* from an external clock signal (*CLK_EXT_*). The CMP compares *V_out_* and a pre-defined DC reference voltage (*V_REF_*). When *V_out_* is lower than *V_REF_*, the CMP’s output equals the supply voltage. In contrast, the CMP’s output equals ground when *V_out_* is higher than *V_REF_*. The edge detection logic passes *CLK_EXT_* only when the CMP’s output equals the supply voltage. As a result, since the SAR ADC only performs sampling and conversion when radiation particles are injected and *V_out_* is near the peak value, the PTSG drastically reduces the averaging power consumption of the SAR ADC by reducing the operation time of the ADC. In addition, the PTSG reduces the power consumption of the data extraction module because our sensor interface does not generate unnecessary data to process and transmit.

When the maximum power consumption of the sensor interface is limited in a mobile dosimeter by battery size, our sensor interface can use a higher *f_s_* than previous sensor interfaces because the operation time of the ADC is reduced. As a result, our sensor interface quantizes the peak value of *V_out_* through high *f*_s_ instead of the shaper, which causes distortion.

Commercial SiPM has various problems to be considered when actually using it [13]. Therefore, it is important to set *V_REF_* in our sensor interface for optimizing the power consumption and accuracy. *V_REF_* can be determined after monitoring *V_out_* or the ADC’s outputs (*D_out_*) with radiation check sources, which are harmless to the human body. In addition, the dark current (or count in [13]) should be considered to ensure more accurate operation. The dark current is the main noise source of a SiPM, which is caused by thermal electrons generated in the active volume [13]. Since this dark current is smaller than the spike generated by radiation particles, to prevent waste of the power by the dark current, *V_REF_* can be determined by monitoring *D_out_* without the check source. That is, when the radiation energy is not changed, *V_REF_* should be decreased until there is no sudden change in the *D_out_*. Since this dark current is a function of the active area and varies from device to device [13], initial calibration is required for each mobile dosimeter.

**Figure 4:**
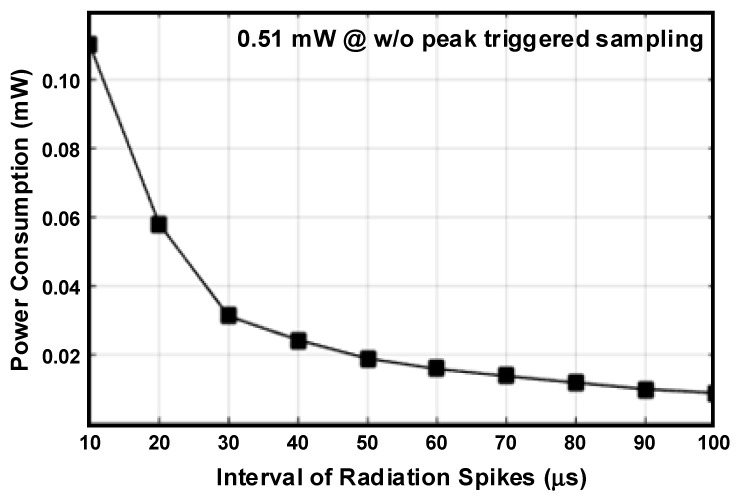
Simulated power consumption of the analog-to-digital converter (ADC) with the peak-triggered sampling scheme according to the interval of radiation spikes.

Figure 4 shows the simulated power consumption of the SAR ADC with the peak-triggered sampling scheme. The power consumption of the SAR ADC is the product of the average current that flows to the circuit and the driving voltage. It can be seen that as the interval of radiation spike increases, it decreases drastically. The DC bias and the peak value of the CSA’s output are about 1 V and 200 mV, respectively. *V_REF_* used in this simulation is 0.6 V, and *f_s_* is 4 MHz. When the interval between radiation spikes is 10 μs, the power consumption of the ADC with the proposed sampling scheme is about 0.11 mW, which is about 21.5% of the ADC’s power consumption when the ADC continuously operates. The power consumption of the ADC decreases as the interval of the radiation spikes increases. When the interval is 100 μs, the power consumption of the ADC with the proposed sampling scheme is reduced to about 0.01 mW.

**Figure 5:**
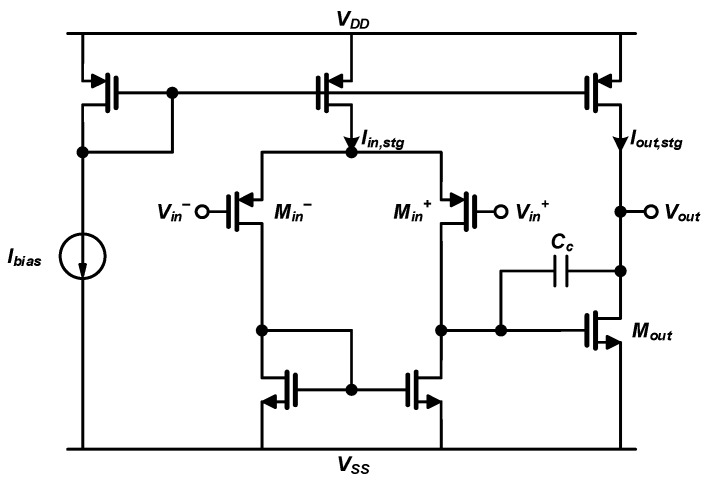
Circuit diagram of the 2-stage operational amplifier (OP-AMP) used in the charge-sensitive amplifier (CSA) and voltage buffer (BUF).

The OP-AMPs of the CSA and BUF are implemented based on the conventional two-stage OP-AMP structure [14], as shown in Figure 5. Each OP-AMP of the CSA and BUF achieves a simulated open-loop bandwidth of 50 MHz with a 5-pF load and a DC gain of 60 dB while consuming only 0.5 mW. These simulated results confirm good enough performances to satisfy the specifications required for the CSA to interface with the SiPM thanks to the open-loop structure of the CSA as well as for the BUF to drive the large sampling capacitor of the following SAR ADC.

**Figure 6:**
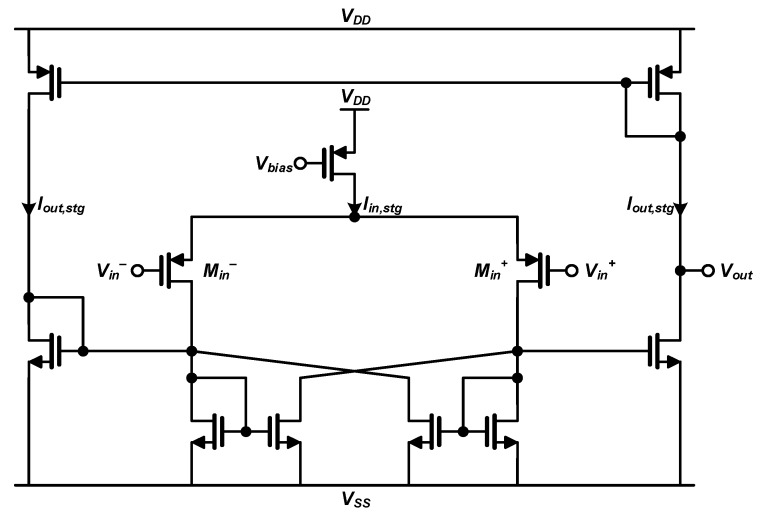
Circuit diagram of the comparator (CMP) used in the proposed peak-triggered signal generator (PTSG).

The CMP of the proposed PTSG has to operate to detect the time moment when the *V_out_* reaches the same value as the *V_REF_*. As shown in Figure 6, the CMP is designed using the cross-coupled hysteresis comparator structure, which is suitable for high-speed operation [15]. Our designed CMP achieves a simulated delay of 10 ns with only 10-μW power consumption. This delay is sufficiently short compared to the time for the CSA’s output to reach the peak value. Furthermore, since the power consumption of the CMP is much smaller than other analog circuits, the power consumption of the overall sensor interface can be minimized.

## 4. Measurement Results

**Figure 7:**
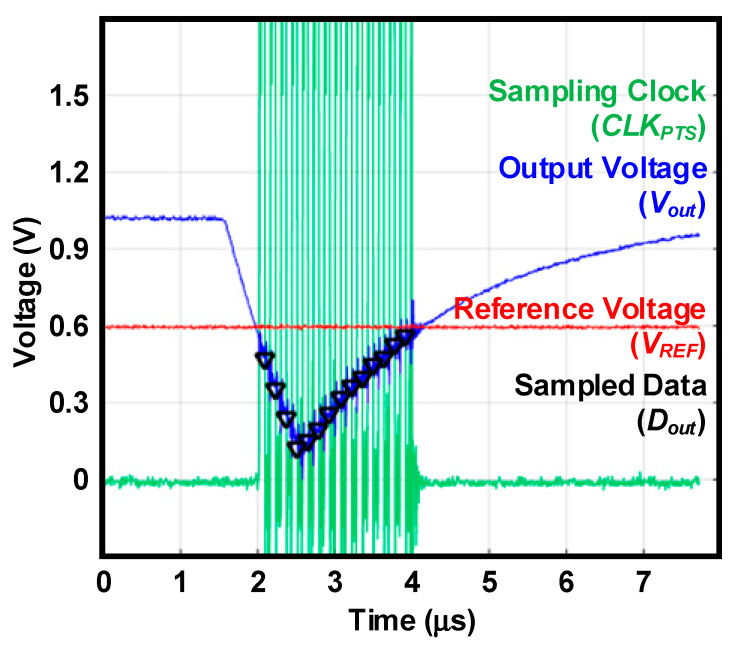
Measured output waveforms with the proposed sampling scheme.

Figure 7 shows the measured output waveforms of the proposed radiation sensor interface. We generate the input charge spike mimicking the condition that SiPM injects 15 fC of charge into the CSA during a sufficiently short time of about 1 μs. When this charge spike is injected, *V_out_* reaches the peak value after 1 μs and recovers to the original DC bias point by *R_fb_*. When *V_out_* is lower than a *V_REF_* of 0.6 V, *CLK_PTS_* exhibits logic transitions with *f_s_* = 4.096 MHz. In contrast, *CLK_PTS_* does not have transitions, and the ADC does not perform sampling and conversion when *V_out_* is higher than a *V_REF_* of 0.6 V. As a result, as shown in Figure 7, the ADC performs sampling and conversion only when a radiation spike is injected and *V_out_* is lower than *V_REF_*. The peak value of *D_out_* shows good agreement with a peak value of *V_out_*.

**Figure 8:**
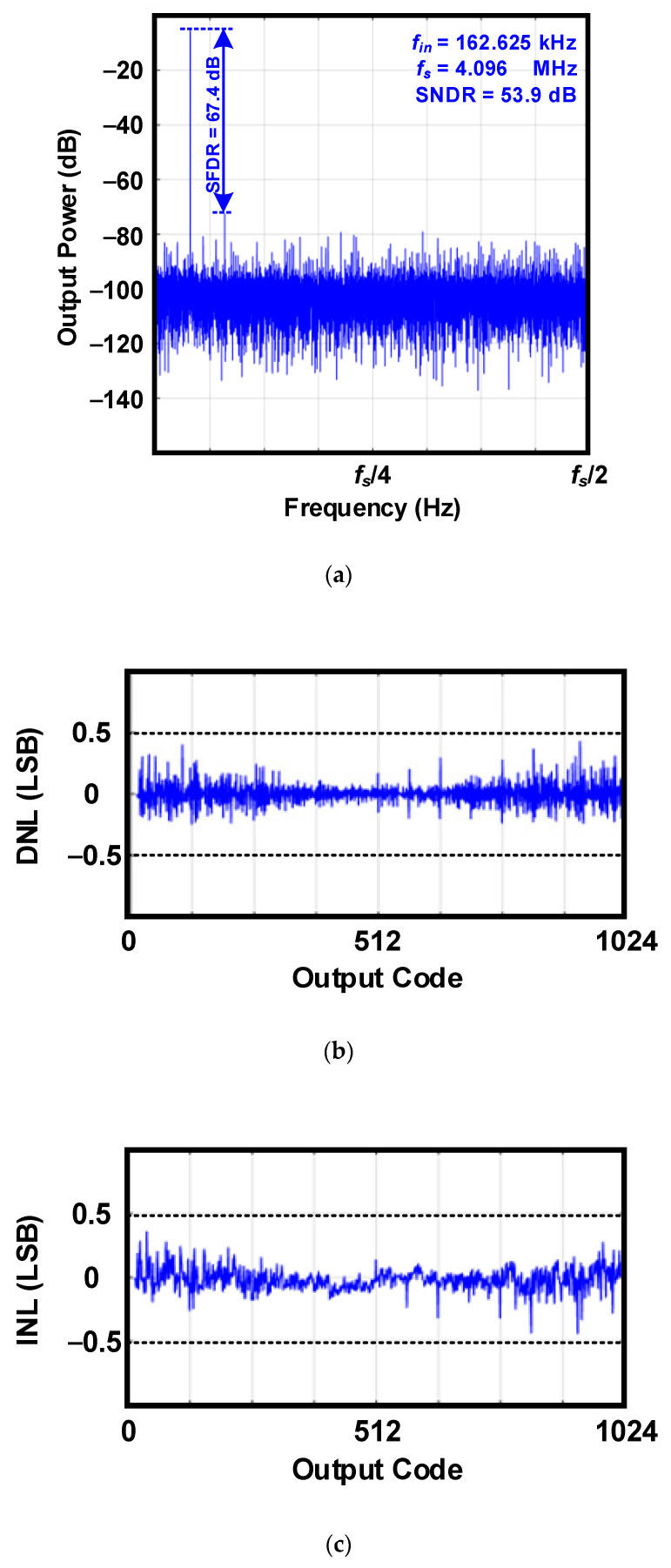
Measured performances of the 10 bit successive approximation register (SAR) ADC: (**a**) Measured output spectrum of digital outputs; (**b**) Measured differential nonlinearity (DNL); (**c**) Measured integral nonlinearity (INL).

Figure 8 presents the measured performances of the 10 bit SAR ADC in order to show how accurately *V_out_* is converted to *D_out_* when *f_s_* = 4.096 MHz. Figure 8a shows the measured output spectrum when a 165.625-kHz sine wave is applied to the SAR ADC. The SAR ADC achieves a signal-to-noise and distortion ratio (SNDR) of 53.9 dB and an effective number of bits (ENOB) of 8.65, which means that the ADC has a resolution of 1.8 V/28.65 = 4.44 mV. In addition, as shown in Figure 8b, the differential nonlinearity (DNL) and integral nonlinearity (INL) are measured below 0.5 least significant bit (LSB). That is, this SAR ADC has sufficiently good linearity. As a result, this designed ADC achieves high resolution and obtains reliable data even at high *f_s_*.

**Figure 9:**
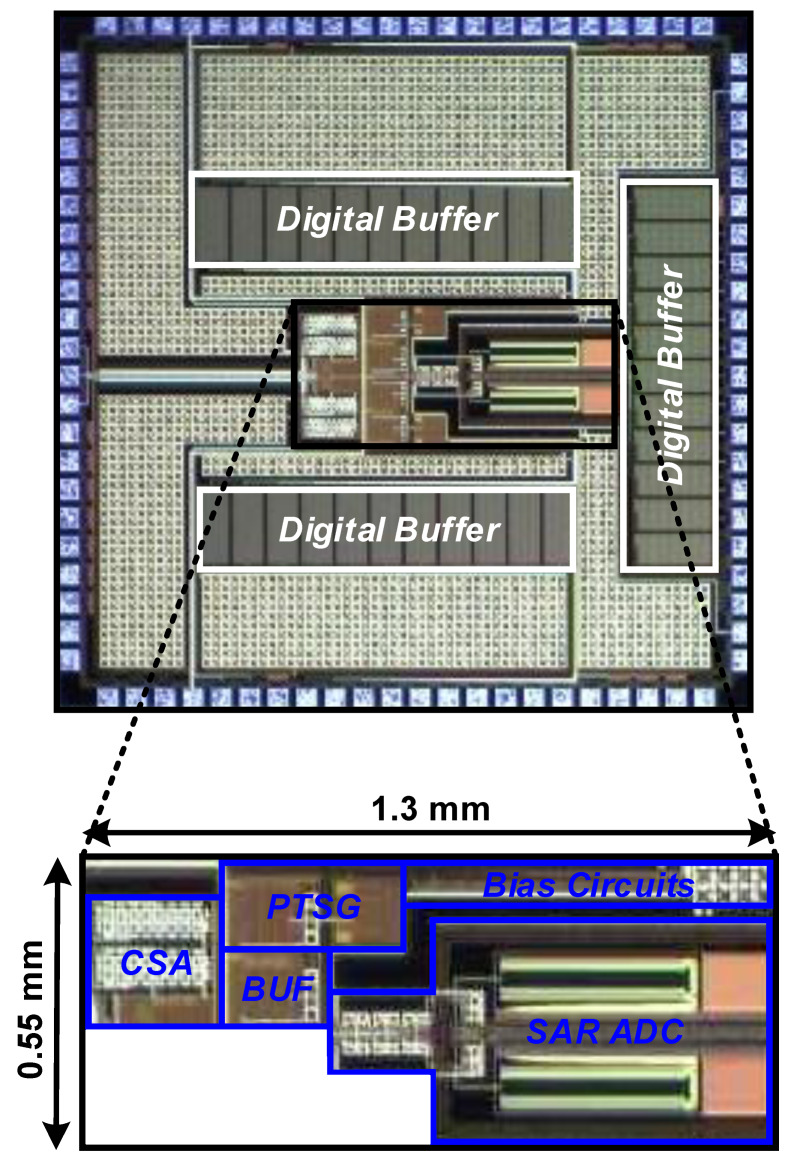
Die photograph of the proposed radiation sensor interface IC.

Figure 9 shows a die photograph of the proposed radiation sensor interface integrated circuit (IC). The proposed sensor interface is implemented in a 180-nm standard CMOS, and the fabricated IC occupies a small area of 0.715 mm^2^. In particular, the proposed PTSG occupies a much smaller area than the SAR ADC, which occupies a dominant portion of the total area.

A comparison of the performance of the proposed sensor interface with prior interfaces is shown in Table 1. The proposed interface for mobile dosimeters includes an ADC, unlike prior interfaces, and its performance is also described. When the ADC continuously operates, the ADC consumes 0.51 mW. However, when the peak-triggered sampling scheme is used, the power consumption of the ADC is 0.11 mW and 0.01 mW for intervals of 10 μs and 100 μs, respectively. The CSA and BUF are implemented with relatively low power consumption.

## 5. Conclusions

We presented a radiation sensor interface using a peak-triggered sampling scheme. The proposed sampling scheme drastically reduces the power consumption of the ADC because the operation time of the ADC is reduced significantly. In addition, when the maximum allowable power consumption is limited, our sensor interface can use a higher *f*_s_ than conventional ones. Therefore, our sensor interface accurately quantizes the peak value of the CSA’s output without a shaper, which causes distortion. As a result, our proposed radiation sensor interface that achieves low power consumption and high accuracy is suitable for mobile dosimeters.

## Figures and Tables

**Table 1:** Performance summary and comparison.

Parameters	[6]	[7]	This Work
Process (nm)	500	350	180
Structure	CSA + Shaper	CSA + Shaper	CSA + BUF + ADC
Area/Ch (mm^2^)	1	N/A	0.715
Power/Ch (mW)	6	1	1 (CSA+BUF) < 0.11 (ADC)
*f_s_* (MHz)	N/A	N/A	4.096
SNDR (dB)	N/A	N/A	53.9
SFDR (dB)	N/A	N/A	67.4
ENOB (bits)	N/A	N/A	8.65
